# Olfactory cues of large carnivores modify red deer behavior and browsing intensity

**DOI:** 10.1093/beheco/arab071

**Published:** 2021-07-05

**Authors:** Suzanne T S van Beeck Calkoen, Rebekka Kreikenbohm, Dries P J Kuijper, Marco Heurich

**Affiliations:** 1 Department of Visitor Management and National Park Monitoring, Bavarian Forest National Park, Freyunger Straβe 2, Grafenau, Germany; 2 Chair of Wildlife Ecology and Management, Albert Ludwigs University Freiburg, Tennenbacher Straβe 4, Freiburg, Germany; 3 Department of wildlife sciences, Faculty of Forest Sciences, Georg-August University Göttingen, Buesgenweg 3, Göttingen, Germany; 4 Faculty of Geoscience and Geography, Georg-August University Göttingen, Goldschmidtstr. 3, Göttingen, Germany; 5 Mammal Research Institute Polish Academy of Sciences, Stoczek 1, 17–230, Białowieza, Poland; 6 Faculty of Applied Ecology, Agricultural Sciences and Biotechnology, Inland Norway University of Applied Sciences, Koppang, Norway

**Keywords:** ambush, cursorial, foraging behavior, olfactory cues, predation risk

## Abstract

This study examined the effect of perceived predation risk imposed by lynx *(Lynx lynx)* and wolf *(Canis lupus)* on red deer *(Cervus elaphus)* foraging behavior under experimental conditions. We hypothesized that in response to large carnivore scent red deer would increase their vigilance, although reducing the frequency and duration of visits to foraging sites. Consequently, browsing intensity on tree saplings was expected to decrease, whereas a higher proportion of more preferred species was expected to be browsed to compensate for higher foraging costs. We expected stronger responses towards the ambush predator lynx, compared with the cursorial predator wolf. These hypotheses were tested in a cafeteria experiment conducted within three red deer enclosures, each containing four experimental plots with olfactory cues of wolf, lynx, cow, and water as control. On each plot, a camera trap was placed and browsing intensity was measured for one consecutive week, repeated three times. Red deer reduced their visitation duration and browsing intensity on plots with large carnivore scent. Despite red deer showing a clear preference for certain tree species, the presence of large carnivore scent did not change selectivity towards different tree species. Contrary to our hypothesis, we found more pronounced effects of wolf (cursorial) compared with lynx (ambush). This study is the first to experimentally assess the perceived risk effects on the red deer foraging behavior of large carnivores differing in hunting modes. Our findings provide insights into the role of olfactory cues in predator–prey interactions and how they can modify fine-scale herbivore–plant interactions.

## Teaser

In this study, we tested whether an ambush (lynx) and a pursuit (wolf) predator influenced behavior and food selection of a large herbivore. We experimentally showed that under perceived predation risk of both lynx and wolf, the large herbivore reduced visitation time, altered visitation duration, and reduced food intake. Differences in behavior and food intake under perceived predation risk of both predators suggests more pronounced risk effects from the pursuit predator.

## INTRODUCTION

Large carnivores can play an important role in structuring ecosystems (Estes et al. 2011). As a result of the direct and indirect effects of large carnivores, ungulate prey species can change their spatial and temporal distributions (Lima and Dill 1990; Kuijper et al. 2013; Latombe et al. 2014; Bonnot et al. 2020) and increase their vigilance (Delm 1990; Brown 1999; Eccard et al. 2015), all of which can modify the impact that ungulates have on the vegetation (Ripple and Beschta 2004; Bubnicki et al. 2019). Because of the complex trade-off ungulates face between food acquisition and minimizing predation risk, antipredator responses of prey are often highly context-dependent. For example, whereas some areas with high perceived risk are avoided, the benefits of high food availability and quality may exceed the costs of predation risk in others (Brown 1992). Moreover, most studies have focused on single predator–single prey relationships, whereas many ecosystems host multiple predator species (Montgomery et al. 2019). With the recent comeback of large carnivores in Europe, potential prey species are more likely to face multiple predators differing in their spatial distribution and hunting mode, thus creating more complex patterns of risk for their prey. How risk effects of large carnivore species differing in hunting mode influence their prey’s foraging decisions, is still a largely unexplored area. A better understanding of these (multiple) predator-prey interactions is essential to predict the ecosystem impact by large carnivores on ungulate communities.

In the case of ungulates, trade-offs between food acquisition and risk avoidance (Brown and Kotler 2004) often result in suboptimal habitat and resource use (Preisser et al. 2005). For example, increased vigilance behavior as a result of an increase in the perceived predation risk may come at the cost of foraging (Lima and Bednekoff 1999; Brown and Kotler 2004). Furthermore, studies conducted in national parks in North America and Africa found that the shifts in habitat use by large ungulates to avoid predation resulted in a lower diet quality (Edwards 1983; Stephens and Peterson 1984; Hernández and Laundré 2005; Barnier et al. 2014). To compensate for these increased costs resulting from risk avoidance, ungulates may increase their selection towards higher-quality plant species. Experimental studies on marsupials showed that trade-offs between food acquisition and risk avoidance affect diet selectivity (McArthur et al. 2012). However, when the benefits of food acquisition outweigh the costs of predation risk or when predation risk refuges are scarce, ungulates may choose to forage in riskier areas (McArthur et al. 2014; Schmidt and Kuijper 2015).

Prey responses to predation risk depend on their ability to recognize the threat and react to cues indicating predator presence (Gaynor et al. 2019). Predator’s scent can be an important cue determining prey behavior (Kats and Dill 1998). In contrast to visual and acoustic cues, olfactory cues persist even after the predator has left and their intensity changes over time, creating a spatially and temporally varying landscape of fear (Bytheway et al. 2013). The predator’s scent provides information on the identity of the predator and on the time elapsed since its presence in the vicinity (Kats and Dill 1998; Apfelbach et al. 2005). The behavioral reaction of prey towards predator scent is influenced by the hunting mode of a predator (Preisser et al. 2007). As ambush predators are relatively sedentary, their olfactory cues should be strongly indicative of their presence creating stronger (perceived) predation risk effects (Preisser et al. 2007). By contrast, cues from actively moving predators, such as cursorial predators, should provide less precise information on their presence creating weaker behavioral responses in prey (Preisser et al. 2007). Despite the fact that the previously cited meta-analyses conducted by Preisser et al. (2007) were dominated by aquatic systems and invertebrates, stronger behavioral responses resulting from ambush versus cursorial predators have also been found in studies of large terrestrial mammals (Atwood et al. 2009; Thaker et al. 2011). Several studies found that prey increased their vigilance behavior in response to olfactory cues from a variety of large carnivore species, but a direct comparison between carnivores differing in hunting mode has rarely been done (Apfelbach et al. 2005; Kuijper et al. 2014; Eccard et al. 2015; Sahlén et al. 2016; Haswell et al. 2018)

This study examined the effects of the scents of an ambush (Eurasian lynx) and cursorial predator (wolf) on ungulate (red deer) foraging behavior, in an experimental design that allowed to control for the effects of other confounding factors. Red deer was studied as it is an important prey species of both wolf and lynx (Okarma et al. 1997; Jędrzejewski et al. 2002). We simulated large-carnivore presence by applying both scat and urine on experimental plots planted with five tree species, which was in our experiment the primary predator cue. Because ungulates foraging under predation risk face a trade-off between food acquisition and predator avoidance, we hypothesized that red deer in the presence of olfactory cues of large carnivores:

1) Increase their time spent vigilant and reduce visitation rate and visitation duration.
2) Resulting in a lower browsing intensity of tree saplings.
3) Show a stronger selection towards more preferred tree species to compensate for the higher costs of perceived predation risk.
4) Show stronger effects on the behavioral response, browsing intensity, and browsing selectivity, indicative of higher perceived predation risk, towards the scent of an ambush predator, compared with a cursorial predator.

This study is the first to compare the effects of the olfactory cues of ambush and cursorial predators on multiple aspects of red deer foraging behavior and diet selectivity. Our results contribute to unraveling the complex interactions between large carnivores and ungulates within European forests.

## METHODS

### Study area

Our study was conducted in three different red-deer enclosures within and surrounding the Bavarian Forest National Park (242 km^2^, 49° 3′ 19″N, 13° 12′ 9″E), situated in southeast Germany. Two of the enclosures are part of the National Park and visitors are allowed entry but restricted to walking paths. The third enclosure is privately owned and visited only by the owner and his family. The size of the different enclosures was 3.8 ha, 7.8 ha, and 1.2 ha containing 16, 14, and 9 red deer individuals, respectively (Table 1). The deer were fed daily with hay, carrots, and pellets (Table 1). Furthermore, in each enclosure no other small tree species were available. Instead, grass meadows were present in all enclosures providing additional food resources during the experiment. All red deer were born in captivity.

**Table 1:** Characteristics of the three red deer enclosures within and surrounding the Bavarian Forest National Park

	Enclosure 1	Enclosure 2	Enclosure 3
Number of red deer	16	14	9
Number of males:females: yearlings	2:14:0	4:10:0	2:4:3
Habitat description	Forest with two smaller clearings	Combination of forest and clearings	Open grassland
Enclosure size (ha)	3.8	7.8	1.2
Available food sources	Pellets, grass, carrots	Hay, grass, carrots	Hay

The Bavarian Forest National Park lies in the temperate climate zone and comprises a variety of forest types, including subalpine forest, mixed mountain forests, and alluvial forest, consisting mainly of Norway spruce (*Picea abies*); European beech (*Fagus sylvatica*), silver fir (*Abies alba*), rowan (*Sorbus aucuparia*), and sycamore maple (*Acer Pseudoplatanus*) (Cailleret et al. 2014). The Eurasian lynx was eradicated from the area in 1848 but reintroduced in the 1970s (Wölfl et al. 2001). Currently, the population is considered to be stable, with an estimated density of approximately 1–2 lynx/100 km^2^ (Heurich et al. 2015; Palmero et al. 2020). In recent years, wolves have been recolonizing the area. In 2016, a wolf pair was documented in the area, and in 2017 the first established wolf pack since 1846 was confirmed. During the time of our study, the exact number of wolves in the area was unknown, with only a few sightings (camera trap data). As both lynx and wolf were able to roam the area surrounding the red deer enclosures, olfactory cues of these large carnivores might have previously been observed by the red deer.

### Experimental design

#### Experimental plots

**Figure 1:**
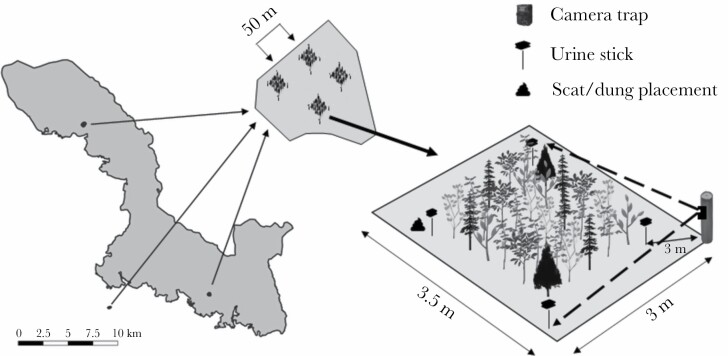
Graphical abstract of the study areas presenting the locations of the three red deer enclosures within and surrounding the Bavarian Forest National Park and the experimental plot design within each enclosure.

The effects of predation risk on red-deer diet selection and vigilance behavior were investigated in a cafeteria experiment conducted within each of the three enclosures (Figure 1). Olfactory cues of wolf and lynx were used to represent cursorial- and ambush predators, respectively, and those of cow to control for unknown, nonhazardous smells. Water served as a control for the possible presence of human smell after cue placement. Within each enclosure, four experimental plots, each with an area of approximately 3.5 m × 3 m, were set-up (Figure 1). Each experimental plot contained one of the following treatments: wolf urine/scat, lynx urine/scat, cow urine/dung, and the control water treatment. Wolf and lynx urine samples were purchased online (www.predatorpee.com; Maine Outdoor Solutions, 2706 Union St., Hermon, Maine 05501 USA). Urine of Eurasian lynx was not for sale, therefore, we had to purchase bobcat urine (*Lynx rufus*) instead. As these two species are from the same genus, we do not expect that this influences our results. Last, cow urine was collected by the owners of a dairy farm. All urine samples were stored in a refrigerator at approximately 6 °C until needed. Wolf scat, lynx scat, and cow dung were collected from the respective animal enclosures within the national park and stored frozen at −20 °C.

The four experimental plots were separated from one another by a minimum distance of 50 m to minimize airborne scent contamination (demonstrated in Kuijper et al. 2014; Wikenros et al. 2015). The experiment was conducted between 08.04.2019 and 28.06.2019, during which time all four treatments were repeated three times within each of the three enclosures. Within each repetition round, measurements (red deer behavioral response and browsing measurements) were conducted during one consecutive week, with a 1-week pause between each repetition. As previous studies showed that ungulates respond to a smell during the first 5–7 days after placement (Kuijper et al. 2014; Wikenros et al. 2015) and all scent objects were removed after each experimental period, we assumed that this 1-week interval between measurements was enough to remove the scent from the plot and to minimize carry-over effects. With respect to the plot placement, two of the three enclosures were large enough to allow the four experimental plots to be set up at the same time. For each repetition, experimental plots were set-up at the same location within the red deer enclosures, however, the treatment applied to each plot was randomly selected. In the third enclosure, because only two experimental plots could be established simultaneously, a control plot (water or cow) was always compared with a plot with large carnivore scent. After a 1-week interval, the remaining two scent treatments were randomly applied to two newly planted plots. At the end of the experiment, measurements were conducted on a total of 12 plots per enclosure and a total of 36 plots for the three enclosures.

On the first day of each repetition, a total of 30 trees from five tree species were planted on each experimental plot. These tree species are commonly found in the area of the Bavarian Forest National Park that surrounds the enclosures and differ in their preference by red deer (based on browsing intensity measures found in Möst et al. 2015): Norway spruce, silver fir, European beech, rowan, and sycamore maple. The trees were bought from the same tree nursery and had an average height of 45 (±15.5) cm. For each of the five tree species, six saplings were planted across an equally spaced grid within a plot and separated by a distance of 50 cm from the next sapling (after Churski et al. 2017). Before planting, all saplings were carefully checked and only saplings without any broken shoots were planted to ensure that all damages measured were caused by red deer browsing. The five species were planted pseudorandomly throughout the grid, with not more than two individuals of the same species planted next to each other. This was done to minimize clustering effects by the preferred tree species. Besides the presence of other food sources during the experiment (grass patches, supplementary feeding), no other small trees were available to the deer during the experiment. At each corner, at 50 cm away from each plot, a stick with a sponge mounted on top was placed for the application of the urine (Figure 1). A plastic roof protected the sponge to reduce the effect of rain. Peters and Mech (1975) estimated that wolf scent-marking under natural conditions contains approximately 5 ml of urine. In their study of European lynx, Eccard et al. (2015) found an effect on roe deer (*Capreolus capreolus*) vigilance behavior using 7 ml of urine. To ensure an effect on red deer behavior, we used a total of 12 ml of wolf/lynx/cow urine at each plot (3 ml/sponge) in combination with feces of the respective species. We did this because several studies suggested that a combination of urine and scats create stronger perceived risk effects (Shrader et al. 2008; Kluever et al. 2009; Chamaillé-Jammes et al. 2014; van Ginkel et al. 2019). Evenly sized scat or dung was placed next to one of the urine sticks. On each experimental plot, red-deer behavior was recorded using a camera trap (Cuddeback C123 with infrared flash) placed in the northern direction directly facing the planted trees and the scat location (Figure 1). To ensure that their detection range covered the entire plot, the camera traps were placed 3 m from the plot. On animal detection, the camera traps recorded 30-s videos, both day and night. During set-up of new experimental plots (newly planted by trees), red deer were excluded from the plots using warning tape (effectiveness tested by camera traps). This was removed after all experimental plots within the enclosure were planted, which marked the start of the experiment. At the end of the week, all scent-objects, trees, and camera traps were removed for the 1-week interval period.

#### Quantifying the red deer behavioral response

As red deer move as a group, such that the foraging behavior of one deer is not independent of that of another, only the behavior of a single individual from the group was classified. In some enclosures, the camera could detect an animal >20 m away from the plot. To measure a treatment’s influence within a plot, only individuals within that plot or within a 5-m buffer were analyzed. The selected red deer individual was the one whose behavior was visible for the longest time, not blocked by other individuals, and so forth, and quantifiable. For each deer individual recorded, its vigilance, walking, running, foraging, and sudden rush behaviors displayed within the 30-s video were classified in terms of their duration. Vigilance has been defined as the time that an animal spends scanning its surroundings using its visual, hearing, and/or olfactory modalities, evidenced as the head held in an upright position and without foraging (Lima 1987). Foraging was defined in this study as an animal that was either grazing, browsing, or searching for food. A sudden rush consisted of a shift from standing still to running within 1 s. Behaviors that could not be fully observed (for example, the head of the individual was not visible, or during the night) were recorded as “indefinable.” All other behaviors that did not align with one of the above classifications were quantified as “other behavior.” Visitation duration was defined as the total time the individual was detected in each video (max. 30 s/video). To ensure data independence, all videos recorded within a 5-min interval were considered as single visitation events and consequently combined. The time spent vigilant (s) and the visitation duration (the time the red deer spent on each plot, in seconds) were summed for each of these visitation events. Furthermore, visitation frequency (how often the red deer visited each plot) was calculated by summing the different camera events for each plot.

#### Quantifying browsing intensity and tree selectivity

For each tree sapling within a plot, the amount of browsing was measured twice a day by recording whether: 1) the current year’s apical shoot had been browsed or not (apical browsing) and 2) the proportion (number) of the top 10 lateral shoots browsed (lateral browsing, after Kuijper et al. (2013). The shoot was identified as browsed when the shoot’s tip was missing. If <10 lateral shoots were available for assessment, the maximum available number of lateral shoots was recorded. Browsing intensity was determined based on the summed total number of shoots browsed (lateral and apical) from the total number of available shoots measured per tree (maximum: 11). In addition, tree species and tree height were recorded for each tree sapling.

To test whether the food plants selected by red deer changed under perceived predation risk, tree species were divided into “preferred” and “less preferred” groups a posteriori. In this study, browsing intensity measurements rather than a commonly used selectivity index (that is, Jacob’s selectivity index) were used to determine the preferred and less preferred tree species groups. This was possible because the number of tree saplings presented to the deer in the experiment was the same for all tree species, allowing selectivity to be determined directly based on browsing intensity, without correcting for differences in availability (as required in selectivity indices). Based on the results of a large-scale browsing survey conducted within the Bavarian Forest National Park (Möst et al. 2015), European beech was chosen as a neutrally selected species and a preferred (less preferred) tree species was defined as one with a higher (lower) browsing intensity than European beech. Consequently, European beech was not included in either of the preference groups and was excluded from the browsing selectivity analyses.

#### Ethics statement

Permissions to carry out this study within the animal enclosures within and surrounding the Bavarian Forest National Park were granted by the National Park administration and the owners of the red deer enclosures. Our measurements made use of nondestructive methods and thus did not require further permission. The owners of the wolf and lynx enclosures had all required permissions to keep the animals, and scat collection occurred without disturbance of the animals and in the presence of their caretakers. As in van Ginkel et al. (2019), wolf and lynx urine was purchased from a company that collects urine from animals in game farms, zoos, and preserves (www.predatorpee.com; Maine Outdoor Solutions, 2706 Union St., Hermon, Maine 05501 USA). The urine is collected noninvasively, via a floor drainage system. These facilities meet all health and treatment standards, determined during regular inspections conducted by the responsible state agency. Cow urine was collected on a dairy farm during milking.

## STATISTICAL ANALYSES

### Is foraging behavior influenced by aging scent?

First, we tested whether the amount of urine and the scat applied at the experimental plots was sufficient to provoke a red deer response throughout the experimental period (1 week). If the urine and scat applied would be sufficient, we expected that the time spent vigilant would not linearly decrease, and browsing intensity would linearly increase with time since the start of the experiment. To test this, two models were created, one with the proportion of time spent vigilant and the other with the proportion of shoots browsed (browsing intensity) as dependent variables. For each model, a generalized linear model (GLM) with a β-binomial family (“logit” link function) and a zero-inflation structure was used (glmmTMB package; Brooks et al. 2017). The number of hours since the start of the experiment (time of set-up) was calculated for each browsing measurement and each deer visitation event for each consecutive week. Subsequently, in each of the two models, the interaction between the time since the start of the experiment and treatment was added as the independent variable. Last, as the different enclosures and repetitions have only three different levels each, these were added as independent fixed effects in the model (Bolker et al. 2009). Here we found that neither the time spent vigilant nor red deer browsing intensity was influenced by the interaction between the time since the start of the experiment and the different treatments (model output results summarized in the Supplementary Material; Supplementary Table S1). Thus, aging olfactory cues had no effect on red deer vigilance behavior and browsing intensity. We, therefore, used the browsing measurements obtained at the end of the week for further analysis, as they best reflected overall browsing intensity for each treatment.

### Red deer behavioral responses, browsing intensity, and selectivity with and without large carnivore scent

To test whether foraging behavior differed between treatments, models were created to test the behavioral responses of red deer, browsing intensity, and browsing selectivity (Table 2). Within each of these models, each treatment was compared directly to the control treatment (water). Subsequently, post hoc analyses using pairwise comparisons were conducted for each model using the “emmeans” package (Lenth et al. 2020). Depending on the link function applied in the model, pairwise comparisons were performed on a log scale or log-odds ratio scale.

**Table 2:** Characteristics of the statistical models used for the analyses of red deer behavioral response, browsing intensity, and browsing selectivity under perceived predation risk. All generalized linear regression analyses were conducted using the “glmmTMB” package (Brooks et al. 2017). Cbind functions were added to account for the difference in the total time spent on the plots or the total number of shoots available for each tree species

		Dependent variable	Independent variables	Family
Behavioral response	Time spent vigilant	Cbind(Time_vigilant, visitation_duration – Time_vigilant)	Treatment + Repetition + Enclosure	Zero-inflated Betabinomial (link = “logit”)
	Visitation frequency	Number of events	Treatment + Repetition + Enclosure	Negative binomial (link = “log”)
	Visitation duration	Visitation duration	Treatment + Repetition + Enclosure	Truncated negative binomial (link = “log”)
Browsing intensity		Cbind(Total_browsed, total_available – Total_browsed)	Treatment + Tree species + Tree height + Repetition + Enclosure	Betabinomial (link = “logit”)
Browsing selectivity	Preferred tree species	Cbind(Total_browsed_preferred, total_available_preferred – Total_browsed_preferred)	Treatment + Repetition + Enclosure	Zero-inflated Betabinomial (link = “logit”)
	Less preferred tree species	Cbind(Total_browsed_lesspreferred, total_available_lesspreferred – Total_browsed_lesspreferred)	Treatment + Repetition + Enclosure	Zero-inflated Betabinomial (link = “logit”)

### Behavioral response

Three different models were created to test whether red deer increased the time spent vigilant and reduced visitation frequency and visitation duration on plots with carnivore scent (hypothesis 1; Table 2). Within each of the three models, the different treatments, the repetitions (3 times), and the three different enclosures were added as independent variables (Bolker et al. 2009). The effect of treatment on the proportion of time spent vigilant was tested using a generalized linear model (GLM) with a β-binomial family (“logit” link function) and a zero-inflation structure from the “glmmTMB” package (Brooks et al. 2017) to account for the high number of zero’s. Here, the proportion of time spent vigilant was added as a dependent variable using a cbind function to account for the difference in the total time spent on plots (Table 2). Visitation frequency was determined using the number of events as the dependent variable in a GLM with a negative binomial family (“log” link function). Differences in visitation duration (total time spent on the plot in s) were tested using a truncated negative binomial family (“log” link function; Table 2). Last, for each model pairwise comparisons between treatments, repetitions, and the different enclosures were conducted using the “emmeans” package (Lenth et al. 2020).

### Browsing intensity

To test for an influence of predation risk on red deer browsing intensity (hypothesis 2), a GLM with a β-binomial family (“logit” link function) was used for the proportion of shoots browsed (glmmTMB package; Brooks et al. 2017; Table 2). The proportion of shoots browsed was included as a dependent variable using a cbind function to account for differences in the number of shoots available. The different treatments, tree species, tree height, repetitions, and enclosures were added as independent variables. Similar to the models testing the behavioral response, pairwise comparisons were conducted between treatments, repetitions, and the different enclosures using the “emmeans” package (Lenth et al. 2020).

### Browsing selectivity

To test whether red deer selection towards different tree species changed in the presence of large carnivore scent (hypothesis 3), the tree species were divided into “preferred” and “less preferred” groups using the browsing intensity results on the control plots. To define these groups, a GLM with a β-binomial family (“logit” link function) was used based on the browsing intensity measurements on the control plots only (glmmTMB package; Brooks et al. 2017). The proportion of shoots browsed was added as a dependent variable using a cbind function and the different tree species were added as the independent variable. As stated above (under “Quantifying browsing intensity and tree selectivity”) European beech was chosen as a neutrally selected species. Consequently, the tree species for which the browsing intensity was higher compared with European beech were defined as “preferred” whereas the species with lower browsing intensity as “less preferred.” For each group, a model was created in which the dependent variable was the proportion of shoots browsed (browsing intensity) for the respective tree species using the cbind function (Table 2). A β-binomial family (“logit” link function) was used to test for browsing intensity for each of the groups and a zero-inflated structure from the “glmmTMB” package (Brooks et al. 2017) was added in each model to account for the high number of zero counts. In each model, the different treatments, repetitions, and the enclosures were added as independent variables (Table 2). Pairwise comparisons were conducted between treatments, repetitions, and the different enclosures for each of the two models using the “emmeans” package (Lenth et al. 2020).

All spatial and statistical analyses were conducted in R 3.5.1 (R. Core Team 2020). Residual diagnostics were conducted using the stats (R. Core Team 2020), car (Fox and Weisberg 2019), and DHARMa (Hartig 2020) packages.

## RESULTS

For each of the different treatments between 25–33% of the planted trees was browsed at the end of the experiment (after 3 repetitions) and between 1–2 shoots were browsed on average within a single tree. Throughout the experiment, red deer visited the plots a total of 429 times, with an average visitation duration of 49–83 s per visit, and were vigilant for an average of 4–6% of the total visitation time. Boxplots of raw observational data can be found in the Supplementary material (Supplementary Figures S1–S3).

### Behavioral response towards treatments

The proportion of time spent vigilant (expressed as log-odds) for any of the treatments did not differ from the control treatment (water) (Supplementary Table S2). Furthermore, based on our pairwise comparisons, there were no significant differences between any of the treatments (Supplementary Table S3). We did, however, find differences between the different repetitions and enclosures (Results summarized in the Supplementary Material; Supplementary Table S3).

Visitation frequency was lower on plots with cow treatment (log-odds: −0.438 ± 0.170, *z* = −2.573, *P* = 0.010) and lynx treatment (−0.332 ± 0.167, *z* = −1.988, *P* = 0.047) compared with the water treatment (without scent). Visitation frequency on plots with wolf treatment did not differ from the control treatment (water) (−0.019 ± 0.156, *z* = −0.119, *P* = 0.906; Table 3). The post-hoc multiple comparisons showed a tendency that visitation frequency was higher on plots with the water control treatment compared with plots with cow treatment (log-odds ratio: 1.550 ± 0.264, *t*-ratio = 2.573, *P* = 0.071). Additionally, visitation frequency tended to be lower on plots with cow treatment compared with plots with wolf treatment (log-odds ratio: 0.657 ± 0.112, *t*-ratio = −2.458, *P* = 0.090). Last, we found significant differences between the different repetitions and the different enclosures (Supplementary Table S4).

**Table 3:** Model output results of the generalized linear models predicting the influence of the different treatments, repetitions and enclosures on visitation frequency and visitation duration. To test for differences between treatments, the control plot served as a reference. Significant variables are highlighted in bold (*P* < 0.05)

	Visitation frequency			Visitation duration		
	Estimate ± Std. error	*z*-value	*P*-value	Estimate ± Std. error	*z*-value	*P*-value
(Intercept)	**1.708 ± 0.186**	**9.189**	**<0.001**	**4.938 ± 0.184**	**26.830**	**<0.001**
Cow	**−0.438 ± 0.170**	**−2.573**	**0.010**	**−**0.014 **±** 0.171	**−**0.079	0.937
Lynx	**−0.332 ± 0.167**	**−1.988**	**0.047**	**−0.356 ± 0.166**	**−2.141**	**0.032**
Wolf	**−**0.019 **±** 0.156	**−**0.119	0.906	**−0.453 ± 0.148**	**−3.057**	**<0.001**
Repetition round 2	**0.538 ± 0.152**	**3.540**	**<0.001**	**−0.696 ± 0.156**	**−4.450**	**<0.001**
Repetition round 3	**0.566 ± 0.152**	**3.718**	**<0.001**	**−0.528 ± 0.153**	**−3.461**	**<0.001**
Enclosure 2	**0.344 ± 0.164**	**2.092**	**0.036**	**−0.499 ± 0.173**	**−2.875**	**0.004**
Enclosure 3	**1.022 ± 0.151**	**6.790**	**<0.001**	**−**0.0534 **±** 0.155	**−**0.339	0.735

**Figure 2:**
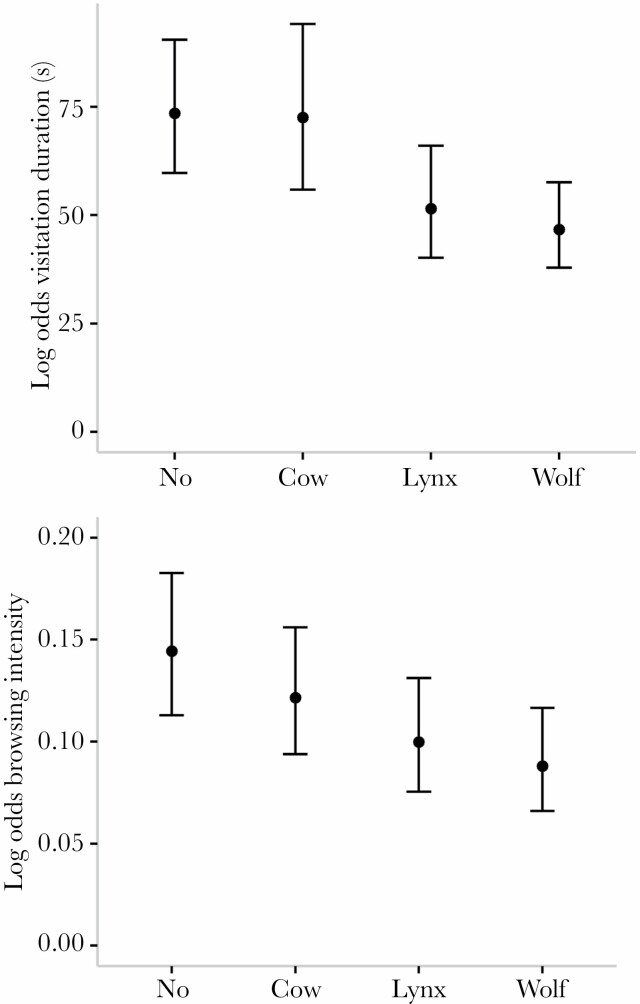
Plots of the generalized linear models showing the log-odds of the visitation duration (s) and browsing intensity (the number of shoots browsed within a single tree) (*y* axis) on plots differing in olfactory cues of wolf, lynx, cow, and water as a control (*x* axis). For each treatment, the fitted values (points) and their 95% confidence intervals are presented (lines).

In contrast to the visitation frequency, we did find that visitation duration was consistently reduced by large carnivore scent (Table 3). The results of our GLM showed that visitation duration was lower on plots with wolf treatment (-0.453 ± 0.148, *z* = 3.057, *P* < 0.001) and lynx treatment (-0.356 ± 0.166, *z* = −2.141, *P* = 0.032) compared with the control (water) (Table 3; Figure 2). Using pairwise comparisons, we found that visitation duration was lower on plots with wolf treatment compared to the nonpredator scent control treatments (water/cow) (log ratio water/wolf: 1.57 ± 0.234, *t*-ratio = 3.057, *P* = 0.013, cow/wolf:1.55 ± 0.266, *t*-ratio = 2.570, *P* = 0.051; Table 5). However, no significant difference was found between plots with lynx compared with all other treatments (Figure 2, Table 5). Significant differences between the repetitions and enclosures were found (Supplementary Table S4).

**Table 5:** Model output results of the post-hoc group comparison tests comparing the visitation duration and browsing intensity between the different treatments. Significant variables are highlighted in bold (*P* <0.05) and variables showing a trend are italicized (*P* < 0.1)

	Visitation duration				Browsing intensity			
	Odds ratio ± Std. error	df	*t*-ratio	*P*-value	Odds ratio ± Std. error	df	*t*-ratio	*P*-value
No/Cow	1.01 ± 0.173	420	0.079	0.999	1.22 ± 0.230	1065	1.050	0.720
No/Lynx	1.43 ± 0.237	420	2.141	0.142	1.52 ± 0.295	1065	2.155	0.137
No/Wolf	**1.57 ± 0.234**	**420**	**3.057**	**0.013**	**1.75 ± 0.340**	**1065**	**2.871**	**0.022**
Cow/Lynx	1.41 ± 0.256	420	1.885	0.236	1.25 ± 0.245	1065	1.121	0.676
Cow/Wolf	*1.55 ± 0.266*	*420*	*2.570*	*0.051*	1.43 ± 0.282	1065	1.829	0.260
Lynx/Wolf	1.10 ± 0.183	420	0.588	0.936	1.15 ± 0.232	1065	0.690	0.901

### Browsing intensity

Browsing intensity was reduced on plots with lynx treatment (−0.419 ± 0.194, *z* = −2.155, *P* = 0.031) and wolf treatment (−0.588 ± 0.194, *z* = −2.871, *P* = 0.004) compared with the control plot (water) (Table 4; Figure 2). However, our pairwise comparisons only showed a significant difference in the log-odds ratio between plots with wolf scent and the nonpredator control treatments (Table 5). Here we found that browsing was 75% higher on the control (water) plots compared with the plots with wolf treatment (log-odds ratio: 1.75 ± 0.340, *t*-ratio = 2.871, *P* = 0.022). Differences in the browsing intensity between tree species were also observed. Compared with European beech, Norway spruce was less intensively browsed, whereas silver fir and rowan had a higher browsing intensity (Table 4). Although browsing on sycamore maple was lower than European beech, this was not statistically significant (Table 4). Furthermore, browsing intensity increased with tree height (0.015 ± 0.007, *z* = 2.297, *P* = 0.022). Additionally, the log-odds ratio of browsing differed between the different repetitions and enclosures (Supplementary Table S5).

**Table 4:** Model output results of the generalized linear model predicting the influence of the different treatments, tree species, tree height, repetitions, and enclosures on the browsing intensity (number of shoots browsed within a single tree). To test for differences between treatments, the control plot served as reference. In tests of the differences between tree species, European beech served as the reference. Significant variables are highlighted in bold (*P*< 0.05) and variables showing a trend are italicized (*P* < 0.1)

	Browsing intensity		
	Estimate ± Std. error	*z*-value	*P*-value
(Intercept)	**−**1.209 ± 0.348	**−**3.478	<0.001
Cow	**−**0.198 ± 0.189	**−**1.050	0.294
Lynx	**−0.419 ± 0.194**	**−2.155**	**0.031**
Wolf	**−0.558 ± 0.194**	**−2.871**	**0.004**
Norway spruce	**−0.741 ± 0.228**	**−3.246**	**0.001**
Rowan	**1.256 ± 0.256**	**4.896**	**<0.001**
Silver fir	**1.038 ± 0.206**	**5.048**	**<0.001**
Sycamore maple	**−**0.364 ± 0.267	**−**1.362	0.173
Tree height	**0.015 ± 0.006**	**2.297**	**0.022**
Repetition round 2	**−2.232 ± 0.200**	**−11.119**	**<0.001**
Repetition round 3	**−0.929 ± 0.157**	**−5.924**	**<0.001**
Enclosure 2	**−1.550 ± 0.199**	**−7.801**	**<0.001**
Enclosure 3	*0.259 ± 0.154*	*1.687*	*0.092*

### Browsing selectivity

Based on the differences in browsing intensity between tree species on the control plots (water), rowan and silver fir were defined as preferred tree species, and sycamore maple and Norway spruce as less preferred tree species. For preferred tree species, browsing intensity was lower on plots with cow treatment (-1.044 ± 0.319, z = −3.274, *P* = 0.001) and wolf treatment (-0.915 ± 0.334, *z* = −2.737, *P* = 0.006) compared with the control treatment (water) (Supplementary Table S6). This is confirmed by our pairwise comparisons, where the browsing intensity was 1.8-fold higher on plots with the control treatment (water) compared with the browsing on plots with cow treatment (log-odds ratio: 2.841 ± 0.906, *t*-ratio = 3.274, *P* = 0.006). Similarly, browsing intensity was 1.5-fold higher on the control plots (water) compared with plots with wolf treatment (log-odds ratio: 2.495 ± 0.834, *t*-ratio = 2.737, *P* = 0.033; Supplementary Table S7). For less preferred tree species, the browsing intensity did not differ from the control treatment for any of the treatments (Supplementary Table S6). Furthermore, based on our pairwise comparisons, none of the log-odds ratios showed a significant difference between any of the treatments (Supplementary Table S7). Additionally, the log-odds ratio of browsing differed between the different repetitions and enclosures (Supplementary Table S7).

## Discussion

This study showed that red deer reduced their visitation duration without increasing their vigilance levels in presence of large carnivore scent, resulting in a lower browsing intensity under perceived predation risk of both lynx and wolf. Even though red deer showed a clear preference for certain tree species, they did not compensate for the reduced food intake in presence of large carnivore scent by selecting a higher proportion of the preferred tree species. In contrast to our hypothesis, we found more pronounced effects caused by wolf scent (cursorial) compared with lynx scent (ambush).

### Behavioral response and browsing intensity towards large carnivore scent

Contrary to our first hypothesis, there was no difference in the time spent vigilant by red deer on plots with lynx or wolf treatment versus those treated with cow scent or the water control. The lower visitation frequency on the lynx plots did not differ from the cow plots, hence, does not provide evidence for a response towards lynx scent. In line with our first hypothesis, red deer spent less time on plots with large-carnivore scent (shorter visitation duration) compared with control and cow plots, indicative of an higher perceived risk on those plots. These results are in line with another study showing that wild roe deer and red deer in the Białowieza Primeval Forest do not change their vigilance but decrease their visitation duration in response to lynx scat (Wikenros et al. 2015). In a study conducted in the same area, Kuijper et al. (2014) found that wolf scat increased the vigilance of red deer but had no effect on the frequency or duration of visitation to the treated plots. The authors attributed these different behavioral responses to different strategies, with avoidance as the best strategy for an ambush predator (lynx) and vigilance the best strategy for a cursorial predator (wolf). Such a difference in the time spent vigilant was not found in this study. Scanning the surroundings is a commonly applied defense strategy and all ungulates must spend a proportion of their time vigilant to minimize the risk of predation. Instead of a treatment effect, we found that the proportion of time spent vigilant was generally higher during the first repetition compared with the second and third repetition. Thus, vigilance behavior seemed to be more triggered by the novelty of a stimulus than the stimulus per se, whereas visitation duration was generally lower on plots with large carnivore scent.

Furthermore, in accordance with our second hypothesis, the presence of large carnivore scent (both lynx and wolf) resulted in a lower browsing intensity compared with the water control plots. Even though browsing intensity was reduced on both these predator scent plots, we only found a significantly lower browsing intensity on wolf plots compared with the water control plots. This is in line with the results found for visitation duration that was also only significantly reduced on the wolf scent plot compared with the nonpredator controls (water, cow). Thus, even though perceived predation risk by lynx seems to influence the behavioral response and browsing intensity of red deer, our pairwise comparisons showed the most pronounced effects in response to wolf scent. This contrasts with our fourth hypothesis, where we expected that the perceived predation risk of an ambush predator would be stronger than to that of a cursorial predator. In general, prey species can recognize the odors of different predator species, allowing for species-specific responses (for a review see; Kats and Dill 1998). Most studies on the differential behavioral responses of prey towards large carnivores have attributed their results to differences in studied predator species’ hunting modes, with stronger antipredator responses triggered by the olfactory cues from an ambush predator than by those from a cursorial predator (Preisser et al. 2007; Wikenros et al. 2015). This is supposed to reflect the more reliable information on predator proximity provided by olfactory cues from an ambush predator. However, our study could not confirm this. Our results could be explained by wolves being the main predator for adult red deer and are rarely predated by lynx (Heurich et al. 2016). In contrast, fawns are generally more vulnerable to lynx predation risk (Heurich et al. 2016). This could explain why olfactory cues from both wolf and lynx influenced the behavioral response and browsing intensity of red deer, with the effects of the wolf being stronger. Last, the visitation duration and browsing intensity did not differ between the water control plots and the plots with cow scent. Even though visitation duration and browsing intensity of red deer were lower on plots with large carnivore scent (wolf or lynx) compared with the plots with cow treatment, these differences were not large enough to be statistically significant. Despite this, the decrease in visitation duration and browsing intensity found on large carnivore plots relative to the control plots (water and cow), and the significant differences found with the water control plots, seems to imply that red deer in this study specifically responded to olfactory cues of large carnivores.

### Food selection under a perceived predation risk

We found that red deer generally preferred to browse on rowan and silver fir, although browsing intensity on all plots was lower on European beech, spruce, and sycamore maple. This is in accordance with several studies showing that silver fir and rowan are highly attractive food sources (Motta 2003; Senn and Suter 2003; Edenius and Ericsson 2015). Our results are also in accordance with those of Möst et al. (2015). In their study, also conducted in the Bavarian Forest National Park, browsing intensity on silver fir and rowan was higher and that on Norway spruce lower than on European beech. Surprisingly, the browsing intensity on sycamore maple in our experiment was low, although this tree is considered a highly preferred species (Seele 2011; Čermák and Grundmann 2014 as cited in; Ohse et al. 2017). This may be due to the small size of the planted trees, as we found that browsing intensity increased with tree height. In this study, browsing intensity was based on a combination of apical shoot browsing and the proportion of the upper 10 lateral shoots browsed. At smaller tree height, sycamore maple contains fewer lateral shoots than other tree species; in our experimental plots, some of these trees had no lateral shoots at all. Even though this was accounted for in the statistical analyses, the low number of shoots available additionally could have led to a lower attractiveness for red deer and thus a generally lower browsing intensity on sycamore maple.

Several studies have shown that a perceived predation risk drives ungulates to shift their habitat use, resulting in a lower-quality diet (Edwards 1983; Stephens and Peterson 1984; Hernández and Laundré 2005; Barnier et al. 2014). However, when forced to forage in risky areas, ungulates may increase their selectivity, increasing foraging on higher-quality plants to compensate for the higher foraging costs (McArthur et al. 2012; McArthur et al. 2014). Accordingly, we expected that red deer would compensate for the loss of foraging opportunities by a stronger preference for higher-quality plants (that is, higher preference). However, we did not find evidence for an increased browsing intensity on the preferred tree species or decrease on the less preferred tree species in the presence of large carnivore scent compared with the control (low risk) treatments. Thus, even though red deer show a clear preference for certain tree species, they do not compensate for the reduced food intake under perceived predation risk by selecting a higher proportion of the preferred tree species. Although the response of foraging animals to predation risk and plant toxin levels has been investigated (McArthur et al. 2014), to our knowledge our study is the first one to test for potential effects of predation risk on tree species selection by ungulate prey.

### Perceived predation risk for red deer in captivity

Conducting this study in red deer enclosures, provided an excellent opportunity to test red deer responses to perceived predation risk without the influence of external environmental effects. However, it has often been suggested that ungulates living in captivity might show a different response to predation risk than the ones living in the wild. Anti-predator behavioral responses can be either innate, that is, genetically hardwired and passed through generations, or acquired properties that are socially transferred or a combination of both (Griffin 2004). Hence, in the absence of large predators for multiple generations or when prey have not coevolved with large carnivores, the antipredator responses of prey may be lost leading to “predator naiveté” (Berger et al. 2001; Apfelbach et al. 2005; Sand et al. 2006; Sih et al. 2010; Carthey et al. 2017). Our finding that the deer altered their behavioral responses and browsing intensity to both lynx and wolf scents indicated that, despite living in enclosures, the studied red deer did not lose their antipredator behavioral response. Although olfactory cues from both lynx and wolf could potentially be present in the direct vicinity of the red deer enclosures, these red deer never faced the direct risk of predation from either one of these large carnivores. The responses measured must therefore reflect innate (genetically hardwired) behavioral antipredator responses. In that case, we would expect even stronger behavioral responses towards large carnivore scent in wild red deer. However, this assumption remains to be tested.

An additional relevant factor leading to a different response of our captive deer compared with wild deer is the ad libitum food that was provided to the first. Within each of the enclosures, alternative food resources were available throughout the study in the form of supplementary feeding and grass patches next to the plots. As a result, the red deer did not need to visit our planted plots to obtain enough food resources and the costs of avoidance of high perceived risk locations were therefore assumed to be low. This likely contrast to wild deer individuals for which avoidance of food rich patches results in a loss of foraging opportunities. However, as no other small tree individuals were available, the tree species planted were highly attractive for the deer. The high attractiveness of the newly planted trees could be observed from the behavioral response and browsing measurements during the first repetition. In general, we observed the longest time spent on each of the plots and higher browsing intensity, during the first repetition compared with the subsequent repetitions. As a result, we argue that the browsing observed reflects the trade-off between foraging and predator avoidance well and is less influenced by the internal state of the animal.

In addition, the differences in behavioral response and browsing intensity between the different enclosures nicely represent the different trade-offs ungulates face when foraging. Due the inconsistent effects found between enclosures (that is, not consistent with differences in habitat, yearling presence), and the fact that all enclosures were subjected to the same external factors (that is, supplementary feeding) we are uncertain as to what might have triggered these differences. For example, visitors and/or the enclosure owners frequently visited each of the enclosures. Here we have to note that the number of visits in each enclosure were not measured and could therefore not be accounted for in our analyses. However, all plots were at the furthest possible distance away from visitor or feeding sites and the behavioral response of red deer was quantified only for individuals on the plot or within a 5-m buffer, which should have minimized the response to visitors. Furthermore, as the results of our behavioral response (time spent vigilant, visitation frequency and duration) do not show the same patterns between the different enclosures throughout the whole experiment, we believe the effects of visitors, if any, were small and cannot explain the differences between enclosures found.

## CONCLUSION

This study is the first to experimentally and simultaneously test the effects of the olfactory cues of two large carnivores differing in their hunting mode on the behavioral responses, browsing intensity, and selectivity of red deer. This study showed that red deer reduced their visitation duration without increasing their vigilance levels in the presence of large carnivore scent, resulting in a lower browsing intensity under perceived predation risk of both lynx and wolf. In contrast to our hypothesis the risk effects imposed by the cursorial predator (wolf) were more pronounced than those from the ambush predator (lynx). This could be explained by wolves more frequently predating red deer compared with lynx. Red deer did not shift their food item selection by selecting a higher proportion of preferred trees species or stronger avoidance of unpreferred trees under higher perceived predation risk.

Our experiment controlled for ecological confounding factors and was thus able to show that the perceived presence of large carnivores influences the foraging behavior of a potential prey species. Differences in the behavioral response and browsing intensity in response to varying levels of predation risk could lead to higher variability in the regeneration (including species composition) of forests. Our research shows that via olfactory cues large carnivores can modify foraging behavior at fine spatial scales that could in the long term have consequences for the impact on woody plant communities and affect the structure and composition of forest ecosystems.

## Supplementary Material

arab071_suppl_Supplementary-Material
