# Supplementary material for: Olfactory cues of large carnivores modify red deer behavior and browsing intensity
Source: Behav Ecol. 2021 Jul 5;32(5):982–92. doi: 10.1093/beheco/arab071 (PMC8528536; doi:10.1093/beheco/arab071)
Supplement: arab071_suppl_Supplementary-Material [file arab071_suppl_supplementary-material.docx]

Exploratory figures


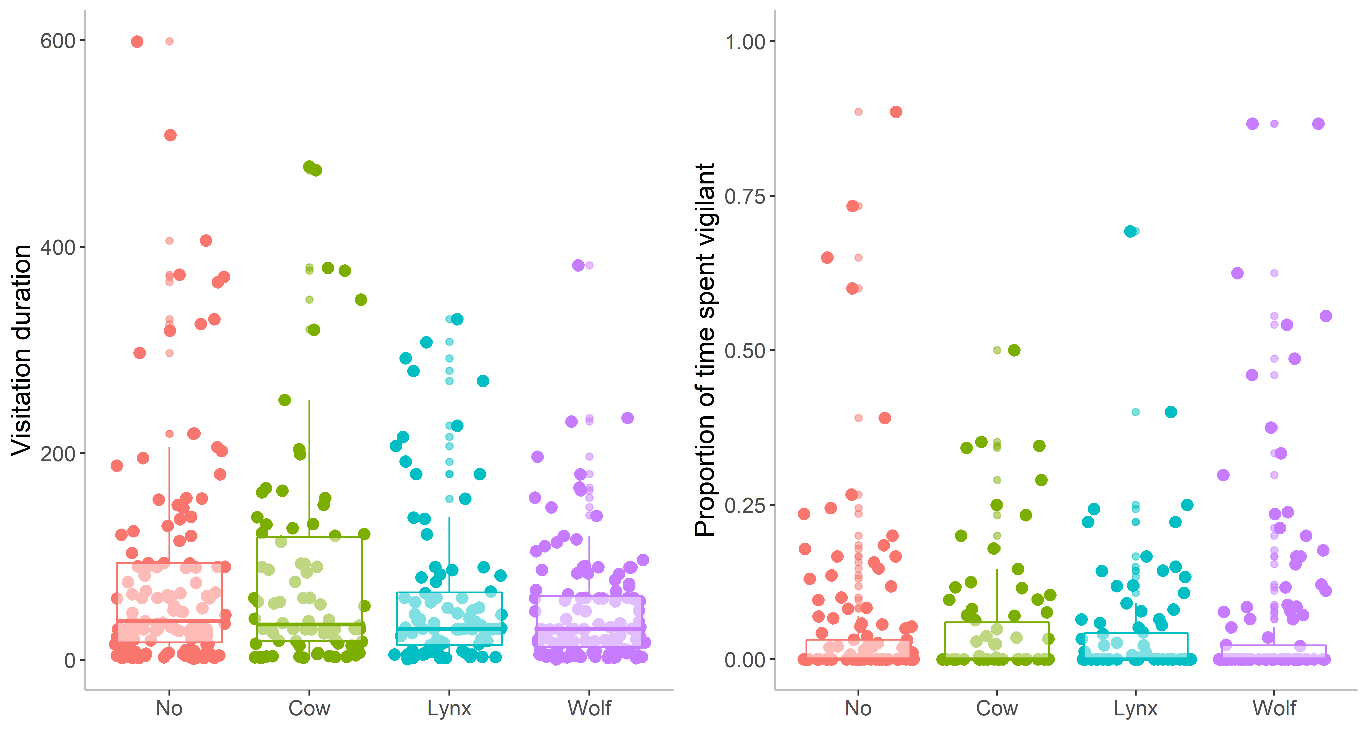
Figure S1: Boxplots of visitation duration and the proportion of time red deer spent vigilant for each of the different treatments (raw observations). The visitation duration follows a truncated negative binomial distribution. whereas the proportion of time spent vigilant follows a betabinomial distribution. In addition, a zero-inflation structure was added within the statistical model for the proportion of time spent vigilant to account for the high number of zero’s.


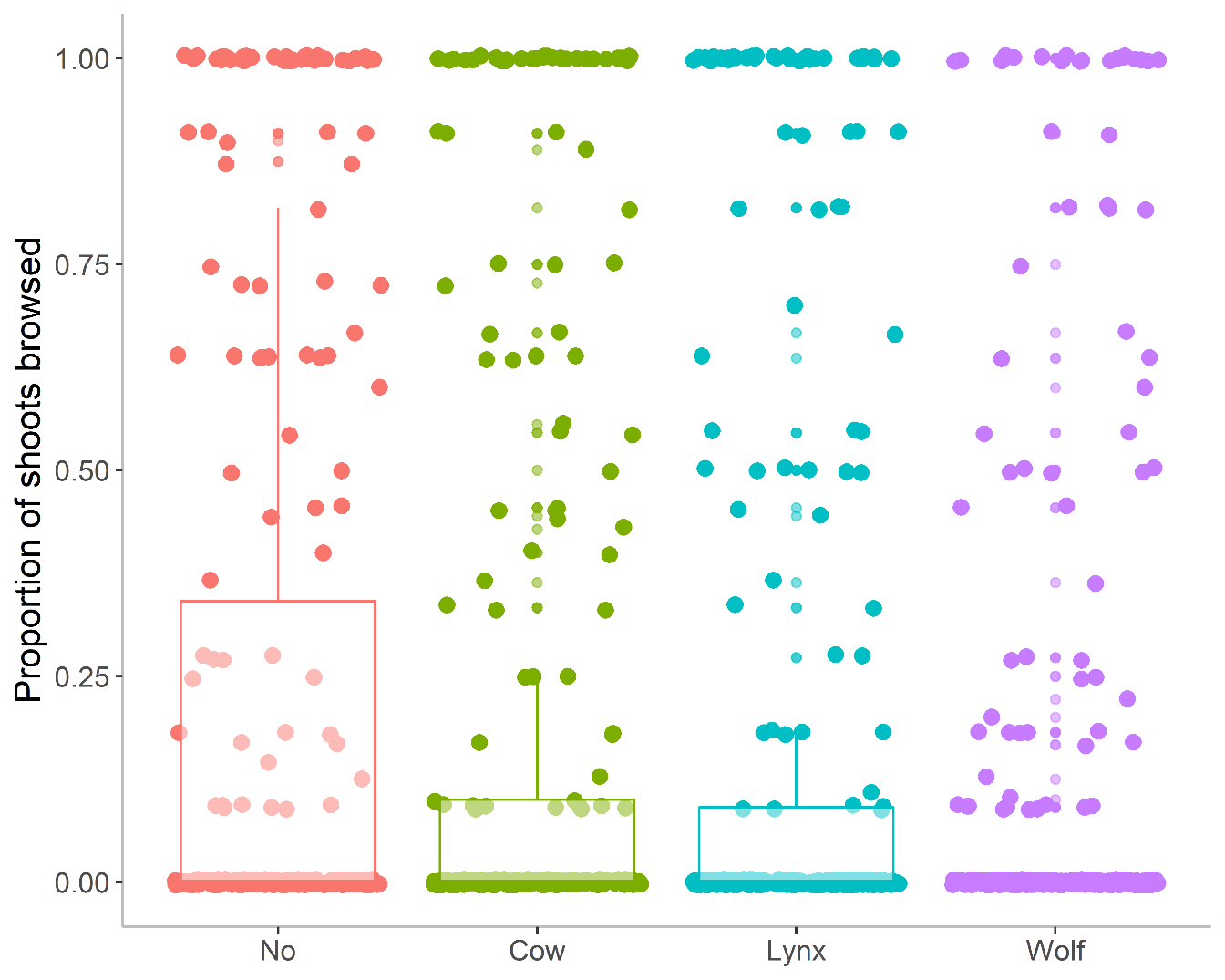
Figure S2: Boxplots of the proportion of shoots browsed for each of the different treatments (raw observations). Here the data follows a betabinomial distribution.


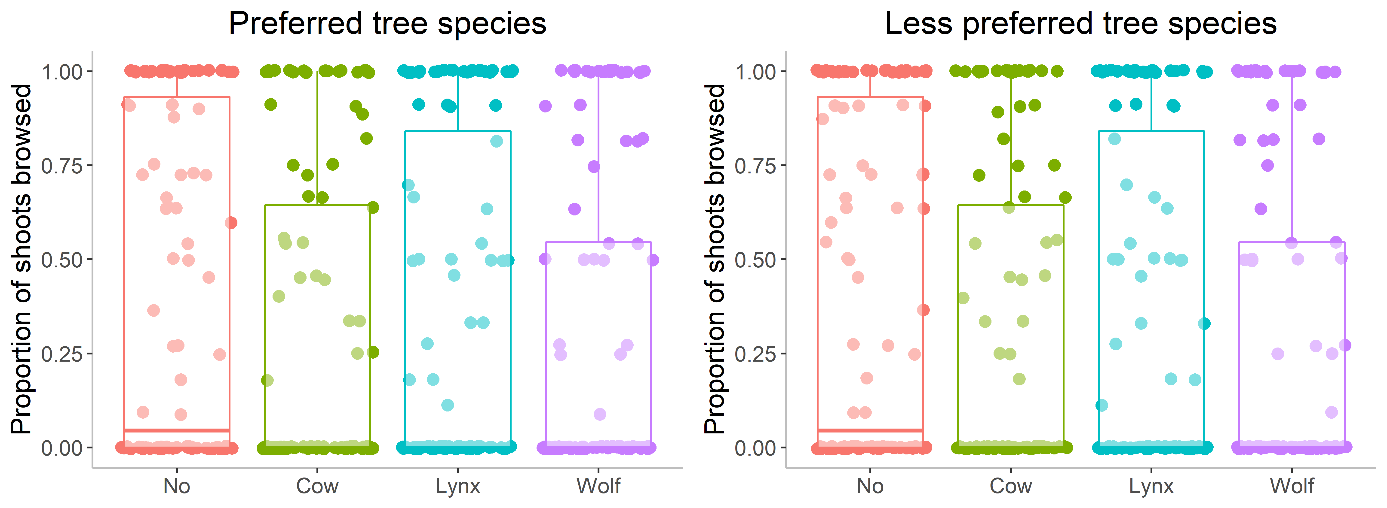
Figure S3: Boxplots of the proportion of shoots browsed on the preferred and less preferred tree species for each of the different treatments (raw observations). Both follow a zero-inflated betabinomial distribution.

Results aging olfactory cues

The time spent vigilant and browsing intensity did not change with the number of hours since the start of the experiment (within one week for each repetition within the different enclosures) and did not differ between treatments (Table S1). Most importantly, we did not find a difference between treatments with time since the start of the experiment (i.e. no significant interactions treatment × time) for the time spent vigilant, nor browsing intensity. In other words, we found no effect of ageing olfactory cues on red deer vigilance behaviour and browsing intensity.

Table S1: Model output results of the generalized linear models predicting the influence of the time since the start of the experiment and the different treatments on the time spent vigilant and browsing intensity (the number of shoots browsed within a single tree) measured throughout one consecutive week. To test the differences between treatments, the control plot served as a reference. Significant variables are highlighted in bold (p<0.05) and variables showing a trend are underlined (p <0.1).

|  | **Time spent vigilant** | | | **Browsing intensity** | | |
| --- | --- | --- | --- | --- | --- | --- |
|  | Estimate ± Std. error | z-value | P-value | Estimate ± Std. error | z-value | P-value |
| (Intercept) | -1.188±0.489 | -2.430 | 0.015 | -2.172±0.268 | -8.109 | <0.001 |
| Cow | 0.652±0.709 | 0.920 | 0.358 | 0.233**±0.361** | 0.644 | 0.519 |
| Lynx | -0.248±0.692 | -0.358 | 0.720 | -0.370±0.380 | -0.973 | 0.330 |
| Wolf | 0.458±0.683 | 0.671 | 0.502 | -0.385±0.380 | -1.014 | 0.310 |
| Repetition round 2 | **-1.031±0.224** | **-4.603** | **<0.001** | **-2.069±0.103** | **-20.065** | **<0.001** |
| Repetition round 3 | **-0.858±0.208** | **-4.120** | **<0.001** | **-0.823±0.068** | **-11.991** | **<0.001** |
| Enclosure 2 | **-0.480±0.242** | **-1.984** | **0.047** | **-1.848±0.121** | **-15.238** | **<0.001** |
| Enclosure 3 | **-0.956±0.207** | **-4.624** | **<0.001** | **0..241±0.074** | **3.247** | **0.001** |
| Cow*Time_since_start | -0.113±0.111 | -1.022 | 0.307 | -0.048±0.049 | -0.985 | 0.325 |
| Lynx*Time_since_start | 0.024±0.106 | 0.226 | 0.822 | 0.002±0.051 | 0.043 | 0.966 |
| Wolf_time_since_start | -0.101±0.103 | -0.985 | 0.325 | -0.030±0.051 | -0.590 | 0.555 |

Results time spent vigilant

Table S2: Model output results of the generalized linear models predicting the influence of the different treatments, repetitions and enclosures on the time spent vigilant. To test for differences between treatments, the control plot served as a reference. Significant variables are highlighted in bold (p<0.05) and variables showing a trend are underlined (p <0.1).

|  | **Time spent vigilant** | | |
| --- | --- | --- | --- |
|  | Estimate ± Std. error | z-value | P-value |
| (Intercept) | -1.443±0.253 | -5.709 | <0.001 |
| Cow | -0.046±0.249 | -0.185 | 0.853 |
| Lynx | -0.099±0.244 | -0.404 | 0.686 |
| Wolf | -0.221±0.241 | -0.920 | 0.358 |
| Repetition round 2 | **-1.070±0.223** | **-4.798** | **<0.001** |
| Repetition round 3 | **-0.883±0.208** | **-4.245** | **<0.001** |
| Enclosure 2 | -0.473±0.241 | -1.965 | 0.050 |
| Enclosure 3 | **-0.939±0.206** | **-4.569** | **<0.001** |

Based on our pairwise comparisons, there were no significant differences between any of the treatments (Table S3). The time spent vigilant was 1.9-fold higher in repetition 1 compared to repetition 2 (log-odds ratio: 2.917±0.650, t-ratio = 4.798, p < 0.001) and 1.4-fold higher compared to repetition 3 (log-odds ratio: 2.419±0.503, t-ratio = 4.245, p<0.001; Table S3). Last, we found that the time spent vigilant was higher in enclosure 1 compared to enclosure 3 (log-odds ratio: 2.56±0.526, t-ratio = 4.569, p <0.001) and tented to be higher in enclosure 2 compared to enclosure 3 (log-odds ratio: 1.59±0.358, t-ratio = 2.080, p =0.095; Table S3).

Table S3: Model output results of the post-hoc group comparison tests comparing the time spent vigilant between the different treatments, repetitions, and enclosures. Significant variables are highlighted in bold (p<0.05) and variables showing a trend are underlined (p <0.1).

|  | **Time spent vigilant** | | | |
| --- | --- | --- | --- | --- |
|  | Odds ratio ± Std. error | df | t-ratio | P-value |
| No/Cow | 1.05±0.260 | 419 | 0.185 | 0.998 |
| No/Lynx | 1.10±0.270 | 419 | 0.404 | 0.978 |
| No/Wolf | 1.25±0.300 | 419 | 0.920 | 0.794 |
| Cow/Lynx | 1.05±0.279 | 419 | 0.199 | 0.997 |
| Cow/Wolf | 1.19±0.314 | 4419 | 0.665 | 0.910 |
| Lynx/Wolf | 1.13±0.293 | 419 | 0.473 | 0.965 |
| Repetition 1/ Repetition 2 | **2.917±0.650** | **419** | **4.798** | **<0.001** |
| Repetition 1/ Repetition/3 | **2.419±0.503** | **419** | **4.245** | **<0.001** |
| Repetition 2/ Repetition 3 | 0.829±0.190 | 419 | -0.818 | 0.692 |
| Enclosure 1/ Enclosure 2 | 1.61±0.387 | 419 | 1.965 | 0.122 |
| Enclosure 1/ Enclosure 3 | **2.56±0.526** | 419 | **4.569** | **<0.001** |
| Enclosure 2/ Enclosure 3 | 1.59±0.358 | 419 | 2.080 | 0.095 |

Results visitation frequency and visitation duration

Visitation frequency was lower in repetition 1 compared to the visitation frequency in repetition 2 (log ratio: 0.584±0.089, t-ratio - -3.540, p = 0.004) and round 3 (log ratio: 0.568±0.087, t-ratio = -3.718, p = 0.003; Table S4). Last, visitation frequency in enclosure 1 was lower compared to enclosure 3 (log ratio: 0.360±0.0542, z = -6.790, p <0.001) and was lower in enclosure 2 compared to enclosure 3 (log ratio: 0.507±0.070, t-ratio = -4.917, p < 0.001; Table S4).

Furthermore, our binary log ratio showed that visitation duration was higher in repetition 1 compared to repetition 2 (log-odds ratio: 2.006±0.314, t-ratio = 3.450, p = 0.001) and round 3 (log-odds ratio: 1.696±0.259, t-ratio = 3.461, p = 0.002; Table S4). Last, the total time spent on a plot was higher in enclosure 1 compared to enclosure 2 (log-odds ratio: 1.65±0.286, t-ratio = 2.875, p = 0.012) and was lower in enclosure 2 compared to enclosure 3 (log-odds ratio: 0.64±0.086, t-ratio = -3.315, p = 0.003; Table S4).

|  | **Visitation frequency** | | | | **Visitation duration** | | | |
| --- | --- | --- | --- | --- | --- | --- | --- | --- |
|  | Log ratio ± Std. error | df | t-ratio | P-value | Odds ratio ± Std. error | df | t-ratio | P-value |
| Repetition 1/ Repetition 2 | **0.584±0.089** | **27** | **-3.540** | **0.004** | **2.006±0.314** | **420** | **4.450** | **<0.001** |
| Repetition 1/ Repetition/3 | **0.568±0.087** | **27** | **-3.718** | **0.003** | **1.696±0.259** | **420** | **3.461** | **0.002** |
| Repetition 2/ Repetition 3 | 0.973±0.134 | 27 | -0.202 | 0.978 | 0.846±0.114 | 420 | -1.242 | 0.429 |
| Enclosure 1/ Enclosure 2 | 0.709±0.117 | 27 | -2.092 | 0.111 | **1.65±0.286** | **420** | **2.875** | **0.012** |
| Enclosure 1/  Enclosure 3 | **0.360±0.0542** | **27** | **-6.790** | **<0.001** | 1.05±0.163 | 420 | 0.339 | 0.939 |
| Enclosure 2/ Enclosure 3 | **0.507±0.070** | **27** | **-4.917** | **<0.001** | **0.64±0.086** | **420** | **-3.315** | **0.003** |

Table S4: Model output results of the post-hoc group comparison tests comparing the visitation frequency and visitation duration between the different repetitions, and enclosures. Significant variables are highlighted in bold (p<0.05) and variables showing a trend are underlined (p <0.1).

Results browsing intensity

Here we found that the browsing intensity was generally lower in repetition 2 compared to repetition 1 and repetition 3 (Table S5). Furthermore, browsing intensity were 1.5-fold higher in repetition 1 compared to repetition 3 (log-odds ratio: 2.532±0.397, z = 5.924, p <0.001). Last, the browsing intensity was higher in enclosure 1 compared to enclosure 2 (log-odds ratio: 4.710±0.936, z = 7.801, p <0.001) and were lower in enclosure 2 compared to enclosure 3 (log-odds ratio: 0.164±0.032, z = -9.140, p <0.001; Figure S5).

|  | **Browsing intensity** | | | |
| --- | --- | --- | --- | --- |
|  | Odds ratio ± Std. error | df | t-ratio | P-value |
| Repetition 1/ Repetition 2 | **9.319±1.871** | **1065** | **11.110** | **<0.001** |
| Repetition 1/ Repetition/3 | **2.532±0.397** | **1065** | **5.924** | **<0.001** |
| Repetition 2/ Repetition 3 | **0.272±0.056** | **1065** | **-6.380** | **<0.001** |
| Enclosure 1/ Enclosure 2 | **4.710±0.936** | **1065** | **7.801** | **<0.001** |
| Enclosure 1/ Enclosure 3 | 0.772±0.119 | 1065 | -1.687 | 0.211 |
| Enclosure 2/ Enclosure 3 | **0.164±0.032** | **1065** | **-9.140** | **<0.001** |

Table S5: Model output results of the post-hoc group comparison tests comparing the browsing intensity between the different repetitions, and enclosures. Significant variables are highlighted in bold (p<0.05) and variables showing a trend are underlined (p <0.1).

Results browsing selectivity

Table S6: Model output results of the generalized linear effect models predicting the influence of the different treatments interacting with the number of repetitions on the browsing intensity (number of shoots browsed from a single tree) for preferred and less preferred tree species. Rowan and silver fir were defined as preferred tree species, and sycamore maple and Norway spruce as less preferred tree species. To test for differences between treatments, the control plot served as a reference. Significant variables are highlighted in bold (p<0.05) and variables showing a trend are underlined (p <0.1).

| **Browsing selectivity** | | | | | | |
| --- | --- | --- | --- | --- | --- | --- |
|  | **Preferred tree species** | | | **Less preferred tree species** | | |
|  | Estimate ± Std. error | z-value | P-value | Estimate ± Std. error | z-value | P-value |
| (Intercept) | 1.259±0.333 | 3.771 | <0.001 | -1.266±0.347 | -3.650 | <0.001 |
| Cow | **-1.044±0.319** | **-3.274** | **0.001** | 0.200±0.385 | 0.521 | 0.602 |
| Lynx | -0.484±0.321 | -1.505 | 0.132 | -9.295±0.418 | -0.705 | 0.481 |
| Wolf | **-0.915±0.334** | **-2.737** | **0.006** | -0.603±0.424 | -1.422 | 0.155 |
| Repetitions round 2 | **-2.370±0.305** | **-7.775** | **<0.001** | **-3.824±0.738** | **-5.179** | **<0.001** |
| Repetition round 3 | **0.298±0.307** | **-6.672** | **<0.001** | **-1.796±0.349** | **-5.153** | **<0.001** |
| Enclosure 2 | **-2.139±0.320** | **-6.672** | **<0.001** | **-1.913±0.569** | **-3.381** | **<0.001** |
| Enclosure 3 | 0.148±0.292 | 0.506 | 0.613 | **0.968±0.317** | **3.055** | **0.002** |

For both preferred and less preferred tree species, browsing intensity was lower in repetition 2 compared to repetition 1 and 3 (Table S7). Last, browsing intensity for the preferred tree and less preferred tree species was higher in enclosure 1 compared to enclosure 2 (preferred log-odds ratio: 8.494±2.724, z = 6.672, p <0.001. ; less preferred log-odds ratio: 6.772±3.832, z = 3.381, p = 0.002; Table S7). Whereas browsing intensity was slightly higher in enclosure 2 compared to enclosure 3 (log-odds ratio: 0.102±0.038, t-ratio = -6.128, p <0.001) for the preferred tree species, browsing intensity for the less preferred tree species was lower in enclosure 2 compared to enclosure 3 (log-odds ratio: 0.056±0.031, t-ratio = -5.195, p <0.001; Table S7).

Table S7: Model output results of the post-hoc group comparison tests comparing the browsing intensity between the different repetitions, and enclosures for the preferred tree species and less preferred tree species groups. Significant variables are highlighted in bold (p<0.05) and variables showing a trend are underlined (p <0.1).

|  | **Browsing selectivity** | | | |  | | | |
| --- | --- | --- | --- | --- | --- | --- | --- | --- |
|  | **Preferred tree species** | | | | **Less preferred tree species** | | | |
|  | Odds ratio ± Std. error | df | t-ratio | P-value | Odds ratio ± Std. error | df | t-ratio | P-value |
| No/Cow | **2.841±0.906** | **422** | **3.274** | **0.006** | 0.818±0.315 | 422 | -0.521 | 0.954 |
| No/Lynx | 1.622±0.521 | 422 | 1.505 | 0.436 | 1.343±0.561 | 422 | 0.705 | 0.895 |
| No/Wolf | **2.495±0.834** | **422** | **2.737** | **0.033** | 1.828±0.776 | 422 | 1.422 | 0.486 |
| Cow/Lynx | 0.571±0.178 | 422 | -1.802 | 0.274 | 1.640±0.682 | 422 | 1.191 | 0.633 |
| Cow/Wolf | 0.878±0.285 | 422 | -0.399 | 0.978 | 2.234±0.941 | 422 | 1.908 | 0.226 |
| Lynx/Wolf | 1.539±0.503 | 422 | 1.318 | 0.552 | 1.362±0.610 | 422 | 0.689 | 0.901 |
| Repetition 1/ Repetition 2 | **10.698±3.261** | **422** | **7.775** | **<0.001** | **45.767±33.786** | **422** | **5.179** | **<0.001** |
| Repetition 1/ Repetition/3 | 0.742±0.228 | 422 | -0.973 | 0.594 | **6.027±2.101** | **422** | **5.153** | **<0.001** |
| Repetition 2/ Repetition 3 | **0.069±0.026** | **422** | **-7.270** | **<0.001** | **0.132±0.102** | **422** | **-2.628** | **0.024** |
| Enclosure 1/ Enclosure 2 | **8.494±2.724** | **422** | **6.672** | **<0.001** | **6.772±3.832** | **422** | **3.381** | **0.002** |
| Enclosure 1/  Enclosure 3 | 0.863±0.252 | 422 | -0.506 | 0.869 | **0.380±0.120** | **422** | **-3.055** | **0.0067** |
| Enclosure 2/ Enclosure 3 | **0.102±0.038** | **422** | **-6.128** | **<0.001** | **0.056±0.031** | **422** | **-5.195** | **<0.001** |
